# Specific isotopic labelling and reverse labelling for protein NMR spectroscopy: using metabolic precursors in sample preparation

**DOI:** 10.1042/BST20210586

**Published:** 2022-11-16

**Authors:** Benjamin Rowlinson, Elodie Crublet, Rime Kerfah, Michael J. Plevin

**Affiliations:** 1 York Structural Biology Laboratory, York Biomedical Research Institute, Department of Biology, University of York, York YO10 5DD, U.K.; 2 NMR-Bio, World Trade Center- 5 Place Robert Schuman, 38025 Grenoble Cedex 1, France

**Keywords:** isotope labelling, NMR spectroscopy, protein

## Abstract

The study of protein structure, dynamics and function by NMR spectroscopy commonly requires samples that have been enriched (‘labelled') with the stable isotopes ^13^C and/or ^15^N. The standard approach is to uniformly label a protein with one or both of these nuclei such that all C and/or N sites are in principle ‘NMR-visible'. NMR spectra of uniformly labelled proteins can be highly complicated and suffer from signal overlap. Moreover, as molecular size increases the linewidths of NMR signals broaden, which decreases sensitivity and causes further spectral congestion. Both effects can limit the type and quality of information available from NMR data. Problems associated with signal overlap and signal broadening can often be alleviated though the use of alternative, non-uniform isotopic labelling patterns. Specific isotopic labelling ‘turns on' signals at selected sites while the rest of the protein is NMR-invisible. Conversely, specific isotopic unlabelling (also called ‘reverse' labelling) ‘turns off' selected signals while the rest of the protein remains NMR-visible. Both approaches can simplify NMR spectra, improve sensitivity, facilitate resonance assignment and permit a range of different NMR strategies when combined with other labelling tools and NMR experiments. Here, we review methods for producing proteins with enrichment of stable NMR-visible isotopes, with particular focus on residue-specific labelling and reverse labelling using *Escherichia coli* expression systems. We also explore how these approaches can aid NMR studies of proteins.

## Introduction

Unlike many other spectroscopic techniques, NMR spectroscopy produces spectra in which it is possible to identify signals that correspond to specific atoms in the target molecule. NMR spectroscopy can, therefore, provide information about molecular structure, dynamics, interactions and biological function at atomic resolution even in molecules as large as proteins or nucleic acids. The full power of the technique is unlocked through the process of resonance assignment: That is, linking a signal in an NMR spectrum to a specific nucleus in the molecule. With assignments in hand, it is possible to determine information about local and global chemistry (i.e. structure, interactions, modifications, etc.) and how this changes with time (i.e. dynamics, kinetics, etc.), and to link this information to prior knowledge of the molecule (e.g. its basic chemical structure, experimental conditions, etc.). Resonance assignment requires being able to resolve individual signals in NMR spectra. Two major issues that complicate the assignment process are signal overlap (resolution) and signal-to-noise ratio (sensitivity). These two factors, which are often linked, can be addressed by combining specialised sample preparation techniques (i.e. isotopic labelling) with appropriate spectroscopic methods.

## Biomolecular NMR spectroscopy and low abundance stable isotopes

Spin-½ nuclei are particularly important for the NMR spectroscopy of biomacromolecules. The ^1^H isotope (natural abundance level: 99.985%) is spin-½, which means that ^1^H NMR spectra can be recorded of proteins without the need for any special isotopic enrichment schemes. However, the dispersion of ^1^H chemical shifts typically found in spectra of proteins is relatively narrow and consequently it is common for ^1^H NMR spectra to suffer from signal overlap. In practice, it becomes challenging to resolve individual ^1^H NMR signals in NMR spectra of proteins larger than 10 kDa.

Spin-½ isotopes of carbon (^13^C) and nitrogen (^15^N) offer improved signal dispersion over ^1^H. However, both have low natural abundance (^13^C at 1.1% and ^15^N at 0.05%) and it is, therefore, necessary to isotopically enrich proteins with these nuclei. Using a protein labelled with ^13^C and/or ^15^N affords better separation of signals by enabling the use of heteronuclear NMR experiments [1]. These experiments can correlate different sets of ^1^H, ^13^C and/or ^15^N nuclei (spin systems) across different dimensions to generate multi-dimensional datasets, which can greatly reduce signal overlap seen in *n*-dimensional ^1^H spectra of proteins [1].

The combination of uniform isotopic labelling of proteins and multi-dimensional heteronuclear experiments is the foundation of modern biomolecular NMR spectroscopy. That said, the application of NMR spectroscopy to proteins is not always straightforward and it is often necessary to overcome significant obstacles.

## Too many signals

NMR spectroscopy can potentially detect signals from each NMR-visible nucleus in a protein, which can mean that even spectra of small proteins will have several hundred observable signals. This problem scales with the size of the protein: As molecular size increases so does the number of observable signals and the likelihood of nuclei having overlapping resonance frequencies.

Size is not the only issue that causes spectral congestion. Many proteins have low or reduced complexity sequences which can lead to signal overlap due to high numbers of residues of the same type or repeats of the same or similar sequence. Transmembrane proteins are often enriched in aliphatic and aromatic amino acids, and regions of NMR spectra where nuclei from these residues resonate can become highly congested [2]. Intrinsically disordered sequences can also present congested NMR spectra due to the lack of chemical shift dispersion that results from secondary and tertiary structure, and that fact that such sequences are often enriched in certain amino acid types and depleted of others [3]. Extreme examples are proteins carrying repeats of certain amino acid types, e.g. poly-glutamine stretches.

## Bigger is not always better

As well as issues relating to signal overlap, NMR spectra of larger proteins also suffer from reduced sensitivity. Larger molecules tumble more slowly in solution, which causes more rapid decay (relaxation) of the NMR signal, broadening of the signal linewidth and concomitant decrease in signal intensity (sensitivity). The principal problem here is that ^1^H nuclei efficiently relax other ^1^H, ^13^C and ^15^N nuclei in their local vicinity. This effect increases with the size of the molecule and becomes highly detrimental for biomolecular NMR studies of proteins larger than 20–25 kDa.

The density of protons in a protein can be reduced by producing the sample in a deuterated expression medium. Deuteration replaces protons with deuterium atoms, which have a smaller impact on the relaxation rates of nearby nuclei [4]. Protein deuteration levels of up to 86% can be achieved using minimal media prepared in deuterium oxide (D_2_O) rather than water [5]. Should higher levels be required then all protons in the culture medium need to be replaced with deuterium, which can be achieved through the use of deuterated carbon sources (e.g. [*U*-^2^H,^13^C] glucose) [5]. While this approach successfully reduces the levels of ^1^H nuclei in proteins, it creates another problem: Protons are commonly used in NMR experiments to excite the system and to detect the final signal, and so retaining some ^1^H nuclei at select sites is beneficial. Protons can be selectively re-introduced into deuterated proteins by exposing the sample to water-based buffers (e.g. during purification) to facilitate the exchange of deuterium for protons in labile sites. Alternatively, protonated (i.e. natural abundance) molecules such as amino acids or metabolic precursors can be added to deuterated expression medium to introduce protons at specific sites in the protein (see below).

Combining deuteration with relaxation-optimised spectroscopic approaches such as TROSY can enable NMR characterisation of much larger proteins and protein assemblies, potentially exceeding MDa sizes (under favourable situations) [6,7].

## Isotopic labelling approaches for protein NMR spectroscopy

Many sample preparation approaches have been developed to alleviate issues of signal overlap and low sensitivity in the NMR spectra of proteins. At the base of most of these is the process of preparing a protein that is uniformly enriched in ^13^C and/or ^15^N nuclei. Protocols for uniform ^13^C and/or ^15^N enrichment of recombinant proteins produced in *Escherichia coli* are well established [8]. The isotopic labelling pattern of a recombinant protein over expressed from *E. coli* can be easily modified by adjusting the labelling pattern of the proton, nitrogen and carbon sources and by supplementing the medium with amino acids or metabolic precursors with the desired level and type of isotopic enrichment. These approaches can be applied to smaller proteins (<15 kDa) by over-expressing from media prepared in water, or to larger proteins (>20 kDa) by over-expression from media prepared with ^2^H_2_O.

### Uniform or site-specific labelling with ^15^N and/or ^13^C

**Figure 1.**
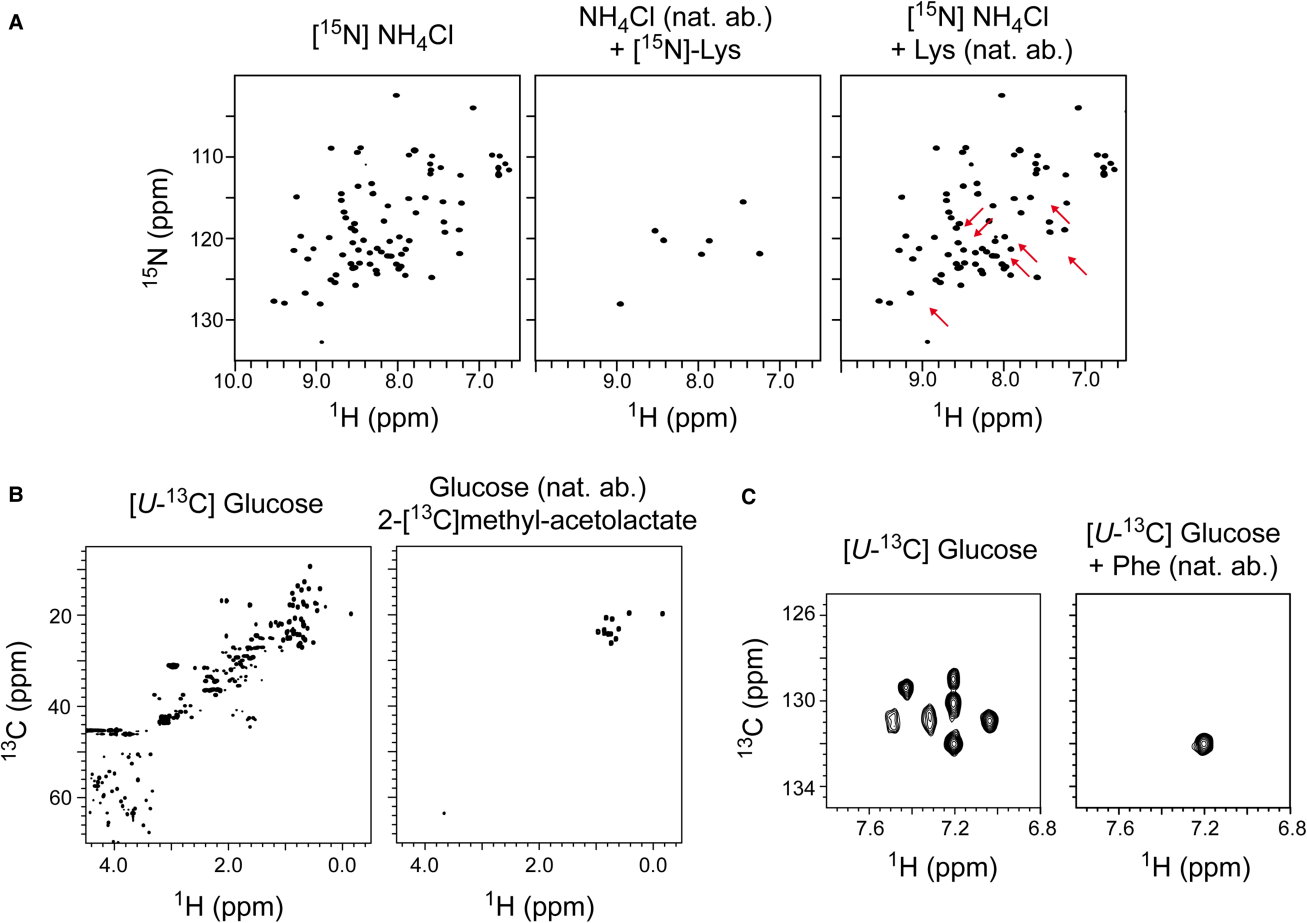
Turning NMR signals on and off using selective labelling or reverse labelling. (**a**) 2D (^1^H,^15^N) HSQC spectra of ubiquitin prepared with [*U*-^15^N] labelled expression medium (uniform labelling; left), unlabelled medium supplemented with [^15^N]-lysine (residue-specific labelling; centre), or [*U*-^15^N] labelled expression medium supplemented with unlabelled lysine (reverse labelling; right; red arrows indicate missing crosspeaks). (**b**) 2D (^1^H,^13^C) HSQC spectra of ubiquitin prepared with [*U*-^13^C] labelled expression medium (uniform labelling; left) or unlabelled medium supplemented with 2-[^13^C]-methyl acetolactate (specific labelling of leucine and valine pro*S* methyl groups; right). (**c**) 2D (^1^H,^13^C) HSQC spectra of ubiquitin prepared with [*U*-^13^C] labelled expression medium (uniform labelling; left) or [*U*-^13^C] labelled expression medium supplemented with unlabelled phenylpyruvate (reverse labelling of phenylalanine; right). Data taken from Rasia et al. 2012 (figure 1A,C) [12] and Gans et al. 2010 (figure 1B) [10].

An expression medium containing ^15^NH_4_Cl as the sole nitrogen source would produce a [*U*-^15^N]-labelled protein, i.e. a protein in which each nitrogen site is ^15^N labelled. A 2D (^1^H,^15^N) HSQC spectrum of such a protein should show a single crosspeak for each residue (excluding proline) as well as crosspeaks for side-chain amine groups (Figure 1a). The enrichment pattern of the protein can be modified by adjusting the composition of the expression medium used to produce it. For example, supplementing unlabelled minimal medium with [*U*-^15^N]-labelled lysine introduces ^15^N label at nitrogen sites in lysine residues [9]. A 2D (^1^H,^15^N) HSQC spectrum of a protein with this labelling pattern would only show crosspeaks for each lysine NH group in the protein and hence be considerably simpler than that of a uniformly labelled sample (Figure 1a). Similar approaches can be considered for metabolic precursors of amino acids.

If a protein is produced using [*U*-^13^C] glucose (CAS number of the unlabelled molecule: 50-99-7) as the sole carbon source, a 2D (^1^H,^13^C) HSQC spectrum should show crosspeaks for each CH group in the protein (Figure 1b). Supplementing an unlabelled expression medium with 2-[^13^C]-methyl acetolactate (CAS: 71698-08-3), which is a precursor in the biosynthesis of leucine and valine [10], produces a protein with [^1^H,^13^C] labelling of pro*S* methyl groups. The resulting 2D (^1^H,^13^C) HSQC spectrum is substantially simplified compared to the uniformly labelled protein as it only shows crosspeaks for pro-*S* methyl groups of leucine and valine (Figure 1b). All other CH sites are not ^13^C labelled and so are not observed in the spectrum.

### Selective reverse labelling of sites in ^15^N and/or ^13^C labelled proteins

An alternative approach is to reverse label specific residues or atoms in an otherwise uniformly labelled protein [11–13]. Supplementing a minimal medium containing ^15^NH_4_Cl with unlabelled lysine produces a protein in which all nitrogen sites are ^15^N labelled except for those in lysine. The resulting 2D (^1^H,^15^N) HSQC spectrum shows that crosspeaks corresponding to lysine residues have disappeared (Figure 1a). Likewise, supplementing ^13^C and/or ^15^N labelled media with unlabelled precursors can turn off signals of specific sets of atoms. For example, adding natural abundance phenylpyruvate (CAS: 114-76-1), a precursor of phenylalanine, to a medium containing [*U*-^13^C]-glucose produces a [^13^C]-labelled protein in which the side-chain groups of phenylalanine are unlabelled [12]. Comparing 2D (^1^H,^13^C) HSQC spectra of uniform and reverse labelled samples show select peaks disappear in the reverse labelled sample (Figure 1c).

## Isotopic labelling using amino acids and metabolites

Specific labelling and reverse labelling of residues and atom subsets is not uniformly applicable. Not every amino acid or precursor will be incorporated ‘as is' into the target protein. Many amino acids and their precursors are metabolised by *E. coli*, which results in the scrambling of the isotopic labelling pattern of the molecule added to the culture medium. There has been considerable research over the last 30–40 years to identify amino acids and metabolic precursors that can be used to manipulate the isotopic enrichment pattern of recombinant proteins overexpressed in *E. coli* with no or minimal isotopic scrambling [4,14,15]. Only certain amino acids are compatible, typically those with an isolated biosynthesis pathway that includes one or more irreversible step. Biosynthetic precursors have emerged as an alternative to using the full amino acid, as precursors often lack stereochemical sites that render the full amino acid expensive to synthesise. Below, we summarise current procedures for residue-specific isotopic labelling and reverse labelling of proteins.

### Routes for targeting aliphatic residues

Aliphatic residues (Leu, Ile, Val and Ala) represent a highly desirable target for specific isotope labelling or reverse labelling due to their relatively high abundance (Leu: 9%, Ile: 5.2%, Val: 6.6% and Ala: 8.3%) and broad distribution across proteins [16,17]. Moreover, these residues contain methyl groups, which are excellent NMR probes for studying larger proteins [18].

Specific labelling and reverse labelling of branched-chain aliphatic amino acids has been used for generating backbone, side-chain and stereospecific assignments as well as for measuring NOEs [4,12,13,19–21].

**Figure 2.**
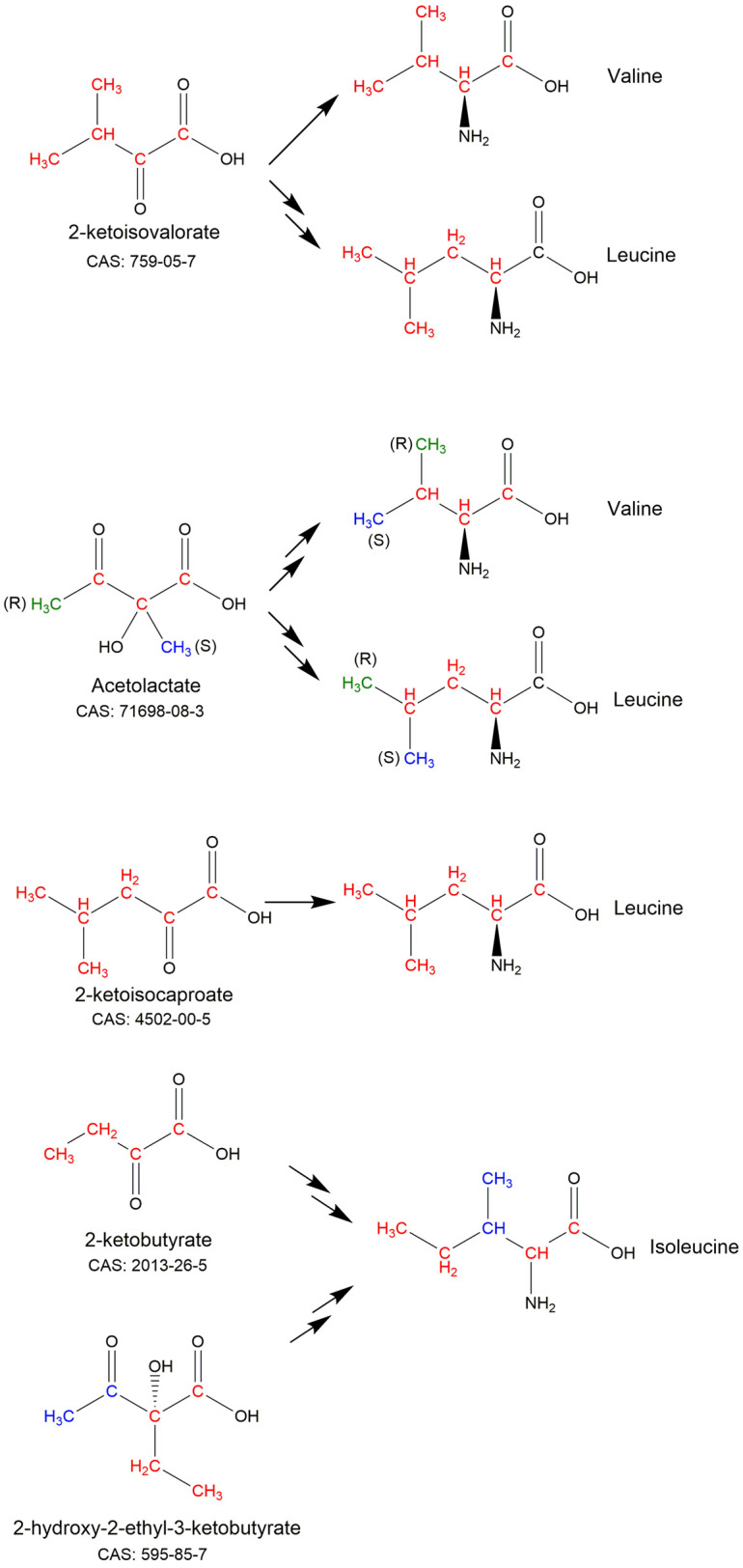
Metabolic precursors that can be used for isotopic labelling and reverse labelling of the carbon sites in branched-chain aliphatic amino acids. Sites corresponding to labelled or unlabelled groups are coloured to show their starting and end positions. The CAS numbers of the unlabelled precursors are given.

The carbon atoms of leucine and valine can be labelled or reverse labelled with minimal scrambling through the use of the biosynthetic precursors α-ketoisovalerate (CAS: 759-05-7) or acetolactate (CAS: 71698-08-3) (Figure 2) [4,10,12,22–24]. Both precursors are chemically synthesised as a racemic mixture, which impacts how they label prochiral methyl groups. Only the 2*S* stereoisomer of acetolactate is a substrate of ketol-acid reductoisomerase (EC: 1.1.1.86), which means that it is possible to use acetolactate to stereospecifically target the prochiral methyl groups of leucine and valine [10,19]. α-ketoisovalerate can be used for applications that require (or can tolerate) labelling of both prochiral methyl groups. Leucine alone can be labelled by the addition of 2-ketoisocaproate (CAS: 4502-00-5) (Figure 2), a precursor of leucine that sits after the divergence of leucine and valine biosynthesis [4,25]. Selectively deuterated versions of these precursors can be used to label larger proteins in combination with deuterated glucose and ^2^H_2_O [10,25].

The carbon atoms of isoleucine can be labelled with 2-ketobutyrate (CAS: 2013-26-5) or 2-hydroxy-2-ethyl-3-ketobutyrate (CAS: 595-85-7) (Figure 2). These molecules also differentially target the methyl groups of Ile: 2-ketobutyrate is used to selectively label the Ile-δ_1_ methyl group, while 2-(S)-hydroxy-2-ethyl-3-ketobutyrate can be used to target Ile-δ_1_ and/or Ile-γ_2_ methyl groups [26–28].

Biosynthetic precursors that target isoleucine, leucine and valine can be used in combination with other metabolites or amino acids which suppress scrambling of carbon sites and off-target effects. For instance, prochiral methyl groups of valine can be selectively labelled by the addition of labelled pro-*R* acetolactate-^13^C_4_ (CAS: 71698-08-3) or pro-*S* acetolactate-^13^C_3_ together with L-Leucine at natural abundance [29]. More details on methyl labelling of isoleucine, leucine and valine can be found in Kerfah et al. 2015 [4] and Schultz and Sprangers 2020 [6].

The methyl group of alanine provides a good probe for monitoring the local structure and dynamics of the protein backbone [30]. Specific isotopic labelling or reverse labelling of alanine is hampered by the presence of alanine transaminases which convert alanine into the widely used metabolite pyruvate [9]. Pyruvate is an early precursor in isoleucine, valine and leucine biosynthesis, which means that the labelling pattern of alanine will be scrambled into other aliphatic residues in the target protein. The addition of 1 g/L natural abundance alanine (CAS: 56-41-7) will result in approximately 50% loss of signal from valine [31]. That said, scramble-free ^13^C labelling of the carbonyl of alanine is possible via the addition of 1-[^13^C]-alanine [32], while labelling of the alanine methyl group can be achieved by adding other metabolites to suppress crosstalk between biosynthesis pathways [33,34]. In principle, reverse labelling can be achieved using similar approaches by adding alanine at the natural abundance and other precursors with ^13^C labelling, though this would be expensive and impractical. [^15^N] labelling or reverse labelling of the backbone amine group of alanine, isoleucine, leucine and valine is problematic due to the action of various transaminases [35].

### Routes for targeting aromatic residues

Aromatic residues are found at interaction interfaces and in the hydrophobic core of proteins and hence can serve as excellent reporters of protein structure, dynamics and interactions [36]. Tryptophan can be used as a sole carbon source by *E. coli* and so significant scrambling of carbon atoms occurs when tryptophan is added to the culture medium [37]. Additionally, tryptophanase (EC:4.1.99.1) can convert tryptophan to indole, pyruvate and ammonia which leads to nitrogen scrambling as ammonia is used in amino acid synthesis [31]. Aromatic amino acid transaminases cause significant nitrogen scrambling between tyrosine and phenylalanine when attempting to label or reverse label with either amino acid [31].

**Figure 3.**
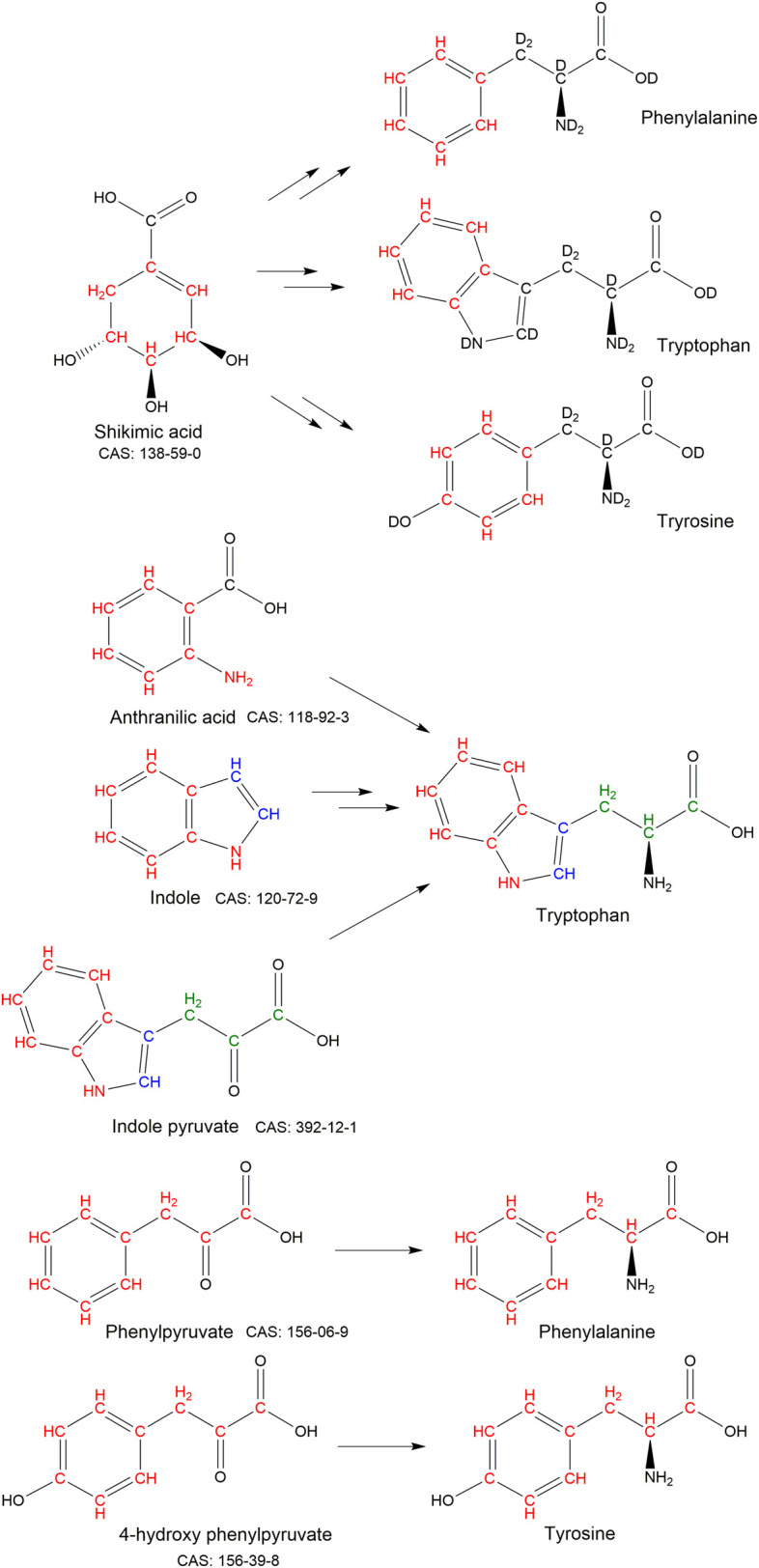
Metabolic precursors used for isotopic labelling and reverse labelling of phenylalanine, tryptophan and tyrosine. Sites corresponding to labelled or unlabelled groups are coloured to show their starting and end positions. The CAS numbers for the unlabelled precursors are given.

An early example of the selective labelling of aromatic residues through metabolic precursors was the use of shikimic acid (CAS: 138-59-0) to label aromatic protons of phenylalanine, tyrosine and tryptophan against a deuterated background (Figure 3) [38]. However, the synthesis of isotopically labelled shikimic acid is complicated, which has precluded widespread use. Phenylpyruvate (CAS: 156-06-9) and 4-hydroxy phenylpyruvate (CAS: 156-39-8) have been used to reverse label the carbon atoms phenylalanine and tyrosine, respectively (Figure 3) [12]. ^13^C labelled versions of these precursors were later reported for isotopic labelling of phenylalanine and tyrosine [39]. Indole (CAS: 120-72-9) can be used for selective tryptophan labelling and reverse labelling of the tryptophan side chain (Figure 3) [40]. Isotopic labelling and reverse labelling of tryptophan can also be achieved through the use of indolepyruvate (CAS: 392-12-1) (Figure 3), which is part of the tryptophan degradation pathway rather than the biosynthesis pathway [41]. Anthranilic acid (CAS: 118-92-3) (Figure 3) has also been reported as an alternative tryptophan labelling precursor, which allows both ^15^N and ^13^C labelling of side-chain sites with minimal scrambling [42].

Histidine can be labelled or reverse labelled by the addition of the amino acid itself to the expression media. Histidine can also be labelled, without scrambling, by the metabolic precursor imidazolepyruvate (CAS: 2504-83-8; Figure 4) [43].

**Figure 4.**
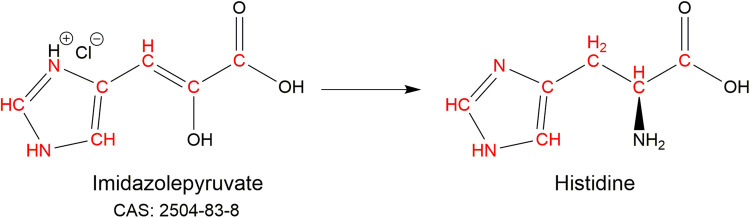
Histidine (un)labelling by the metabolic precursor imidazolepyruvate with incorporated atoms shown in red. The CAS number for the unlabelled precursor is given.

### Routes for targeting polar residues

Serine is connected to glycine via a glycine-hydroxymethyltransferase (EC: 2.1.2.1), which is in turn linked to threonine. Serine is also a precursor of tryptophan and cysteine biosynthesis and can be converted into pyruvate by serine dehydratase (EC: 4.3.1.17). Currently, there is no protocol for scramble-free specific labelling or reverse labelling of serine using traditional expression hosts. Similarly, cysteine is converted to pyruvate by cysteine desulfhydrases (EC: 4.4.1.28) which leads to significant scrambling when cysteine is added to the culture medium.

Threonine is connected to glycine, serine, cysteine, tryptophan and isoleucine, which leads to significant scrambling for nitrogen labelling or reverse labelling [44]. For labelling or reverse labelling of carbon sites, threonine is connected to isoleucine and glycine biosynthesis and can cause significant scrambling when added to the expression media. This scrambling effect has been overcome by the addition of the isoleucine precursor 2-ketobutyrate (CAS: 2013-26-5) (or isoleucine) and glycine to the expression media [44,45].

Asparagine and glutamine are particularly difficult amino acids to specifically label or reverse label. Specific ^15^N labelling of these amino acids has been achieved through the use of media supplemented with ^15^NH_4_Cl and all amino acids at natural abundance apart from asparagine or glutamine [46]. Specific labelling of side-chain sites of these residues using metabolic precursors has not been possible due to their position in metabolic pathways. Asparagine and glutamine synthesis is closely linked to aspartate and glutamate synthesis, which are used in the synthesis of many amino acids. In addition, glutamate is the primary nitrogen donor in amino acid biosynthesis. This means that scramble-free specific labelling of these amino acids with the residues themselves or their precursors is not possible without the addition of a full amino acid complement to the media or the use of auxotrophic strains or cell-free systems [37,47].

### Routes for targeting charged residues

The final steps of both lysine and arginine biosynthesis are irreversible, which means that both amino acids can be used directly for labelling and reverse labelling (Figure 1a), thus negating the need for supplemental precursors to reduce isotopic scrambling.

For reasons discussed above, scrambling free specific labelling of aspartate and glutamate either with the amino acids themselves or with metabolic precursors has not been achieved.

### Special cases

**Figure 5.**
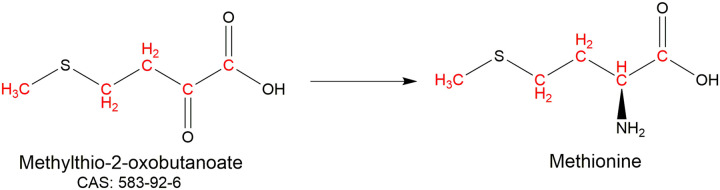
Methionine (un)labelling by methylthio-2-oxobutanoate with isotopically labelled sites indicated in red.

Methionine is commonly used to introduce methyl labelled probes for NMR analyses of proteins [7,48,49]. The relatively low abundance of methionine in proteins (2.4%) can reduce the chance of spectral overlap than with aliphatic residues [17]. Methionine can be both isotopically labelled and reverse labelled with minimal scrambling by the addition of the amino acid itself to the media [31,50]. An alternative approach uses the metabolic precursor methylthio-2-oxobutanoate (CAS: 583-92-6) (Figure 5) for labelling without nitrogen [51].

Glycine is linked to serine and threonine by glycine-hydroxymethyltransferase (EC: 2.1.2.1) and threonine aldolase (EC: 4.1.2.5), respectively, meaning extensive scrambling for both carbon and nitrogen sites occurs when attempting to label or reverse label with glycine.

Proline can be used as a sole carbon and nitrogen source in bacterial cell culture and so induces significant scrambling when added to the media [12,31,37,52]. Metabolic precursors of proline have not been used for the production of proteins with proline labelling or reverse labelling. The interconnectivity of the proline and other amino acid biosynthetic pathways makes this unlikely.

## Discussion

In this review, we have summarised approaches for manipulating the isotopic labelling patterns of recombinant proteins for NMR studies. We have focused on approaches that allow labelling or reverse labelling of specific sets of atoms or residues and discussed how this can be achieved through the addition of amino acids or their precursors to bacterial cell culture media. We have highlighted applications to solution NMR spectroscopy, but the labelling approaches described would also benefit solid-state NMR spectroscopy of proteins.

Extensive research into isotopic labelling protocols means that today's protein NMR scientist can make use of a wide range of labelling and reverse labelling schemes and enrichment patterns. The task now is to utilise these schemes to the greatest effect. In addition to helping with spectral crowding and line broadening, specific isotope labelling or reverse labelling can provide residue-type assignment. These approaches also place site-specific probes into protein that can report on the structure, dynamics and binding (often bypassing the need for full resonance assignment) [12]. For example, a study of the interaction of yeast ubiquitin hydrolase with ubiquitin used a sample prepared with specific ^13^C labelling of Met-Cε, Ala-Cβ, His-Cε, Tyr-Cε and Trp-Cδ and ^15^N labelling of Arg backbone amide groups [53]. Residues involved at the interface were predicted based on the number and type of amino acid chemical shifts that were perturbed on complex formation. These assignment predictions were then used as an input for computational docking using HADDOCK [54] and the resulting models were consistent with a crystal structure of a related complex. The potential of site-specific isotopic labelling or reverse labelling to provide useful information without full assignments can be particularly useful where full assignment may not be possible or be too time consuming to obtain. In the post-AlphaFold2 world [55], lower levels of resonance assignment will likely often be sufficient to confirm a predicted structure or to link protein structure to function [56].

This review has exclusively focussed on the over-expression of recombinant proteins using standard *E. coli*-based techniques, i.e. cytosolic protein production under the control of an IPTG-inducible T7-based expression system. However, not all proteins can be produced using *E. coli*-based approaches and eukaryotic hosts are often required. There has been considerable progress in isotopic labelling using eukaryotic cell types, including yeast, insect and mammalian cells [57–63]. Differences in the metabolic processing of amino acids and their precursors, and different requirements for cell culture media have meant that the production of labelled proteins using eukaryotic systems is still not widely reported in the literature. An overriding concern for isotopic labelling in insect or mammalian cells is the complexity of the cell culture medium compared to the minimal recipes that can be used for *E. coli*. Broadly speaking, modifying eukaryotic cell culture media composition to support isotopic labelling is considerably more expensive than bacterial cell culture. That said, a number of impressive studies have been reported that use eukaryotic expression systems to generate labelled proteins for NMR studies [60,64].

*In vitro* or ‘cell free' protein synthesis is an alternative and highly adaptable approach for producing labelled protein, providing it is compatible with the protein of interest. Working with an S30 cell extract significantly reduces issues from metabolic scrambling compared to protein expression from live cells, and allows residue-specific labelling of each of the 20 proteinogenic amino acids [65,66]. Issues remain with the scrambling of Asp, Asn, Gln and Glu but these can be alleviated through the use of small molecule inhibitors or by using an auxotrophic strain of *E. coli* to produce the cell extract [67,68]. Moreover, the smaller scale of *in vitro* protein synthesis reactions compared to cell culture reduces the costs of reagents. Consequently, the use of amino acids with isotopic enrichment patterns that are complicated or expensive to synthesise becomes feasible because only very low milligram-level quantities are required [69]. Lastly, *in vitro* protein synthesis using amber stop codons and pre-charged tRNA molecules can allow isotopic labelling of a single residue position in the protein of interest [66,70].

NMR spectra can be extremely rich in information. A drawback from selective labelling or reverse labelling of proteins is that it greatly reduces the number of NMR reporters in the target molecule, which can limit some applications and the types of question that can be addressed. Despite that, there are multiple examples of where specific labelling of proteins for NMR studies has considerable benefit over uniform labelling. Ultimately, the isotopic labelling pattern chosen for a particular protein target will depend on the parameters of the system being studied and the biological questions of interest. Many projects do not require a high number of NMR-visible sites or complete resonance assignment to provide relevant information. As NMR spectroscopy moves away from being a method for solving the 3D structures of proteins and embraces a role as a site-resolved spectroscopic technique, quick and efficient access to site-specific NMR probes will become more and more important. Specific isotopic labelling or reverse labelling can provide these important site-specific NMR-visible probes.

## Perspectives

- Uniform enrichment of recombinantly produced samples with ^13^C and/or ^15^N isotopes is a fundamental platform for NMR studies of proteins. Approaches that allow site-specific modulation of the enrichment pattern of a protein can simplify the process of data analysis, provide resonance assignment information and unlock experimental strategies for NMR analysis of structure, dynamics and function.
- Amino acids and/or their biosynthetic precursors can be added to bacterial culture media to achieve site- or residue-specific labelling or reverse labelling of proteins either alone or in combination with standard ^13^C and ^15^N labelling protocols. We review the approaches for achieving different isotopic labelling patterns, outline the motivations for employing these approaches and discuss the experimental benefits for their use.
- Combining experiments that exploit proteins with site-specific labelling patterns with the improved prediction of 3D structure now available via tools such as AlphaFold promises to greatly broaden the range of targets and biological questions that can be addressed by NMR spectroscopy.

## Open Access

Open access for this article was enabled by the participation of the University of York in an all-inclusive *Read & Publish* agreement with Portland Press and the Biochemical Society under a transformative agreement with JISC.
